# Bouveret’s syndrome complicated by iatrogenic esophageal perforation after endoscopic gallstone retrieval: a case report

**DOI:** 10.1093/jscr/rjag173

**Published:** 2026-03-21

**Authors:** Joelle Camilla Korte, Lilly Klingelhöfer, Valerie Nottberg, Jan Bardenhagen, Baris Mercanoglu, Nina Schraps, Thilo Hackert, Nathaniel Melling

**Affiliations:** Department of General, Visceral and Thoracic Surgery, University Medical Center Hamburg-Eppendorf, Martinistraße 52, 20251 Hamburg, Germany; Department of General, Visceral and Thoracic Surgery, University Medical Center Hamburg-Eppendorf, Martinistraße 52, 20251 Hamburg, Germany; Department of General, Visceral and Thoracic Surgery, University Medical Center Hamburg-Eppendorf, Martinistraße 52, 20251 Hamburg, Germany; Department of General, Visceral and Thoracic Surgery, University Medical Center Hamburg-Eppendorf, Martinistraße 52, 20251 Hamburg, Germany; Department of General, Visceral and Thoracic Surgery, University Medical Center Hamburg-Eppendorf, Martinistraße 52, 20251 Hamburg, Germany; Department of General, Visceral and Thoracic Surgery, University Medical Center Hamburg-Eppendorf, Martinistraße 52, 20251 Hamburg, Germany; Department of General, Visceral and Thoracic Surgery, University Medical Center Hamburg-Eppendorf, Martinistraße 52, 20251 Hamburg, Germany; Department of General, Visceral and Thoracic Surgery, University Medical Center Hamburg-Eppendorf, Martinistraße 52, 20251 Hamburg, Germany

**Keywords:** Bouveret’s syndrome, gallstone ileus, esophageal perforation

## Abstract

Bouveret’s syndrome represents a rare variant of gallstone ileus characterized by the migration of a gallstone through a bilioenteric fistula into the duodenum, resulting in pyloric obstruction. We present the case of a 79-year-old woman who presented to a regional hospital with symptoms of vomiting and constipation. Diagnostic imaging revealed duodenal obstruction with multiple concretions and pneumobilia. An attempted endoscopic retrieval of the obstructing gallstones was complicated by an iatrogenic distal esophageal perforation, necessitating transfer to our tertiary care center for further management. Subsequent imaging and endoscopic assessment confirmed the findings and the patient underwent emergency surgical intervention comprising discontinuous resection of the perforated esophageal segment and excision of the cholecystoduodenal fistula. After clinical stabilization, esophageal reconstruction was performed 3 months later via retrosternal colon interposition. The postoperative course was uneventful. This case highlights the necessity of early interdisciplinary management and decision-making in gallstone-induced bowel obstructions and esophageal perforation.

## Introduction

Bouveret’s syndrome is an exceptionally rare cause of gastric outlet obstruction caused by gallstones entering the duodenum via a bilioenteric fistula, representing 1%–3% of all gallstone-related intestinal obstructions [1]. It predominantly affects elderly female patients with chronic gallstone disease, which promotes adhesions and erosions into adjacent gastrointestinal organs, resulting in a bilioenteric fistula [2, 3]. The symptoms are nonspecific, typically including epigastric pain, nausea, and vomiting, making diagnosis challenging [4]. Contrast-enhanced computer tomography (CT) is essential, often revealing Rigler’s triad: pneumobilia, distended stomach, and ectopic gallstones [1]. First-line therapy is primarily endoscopic to minimize surgical risks in elderly, multimorbid patients. If the gallstone is severely impacted and cannot be removed, a surgical approach must be used [5].

## Case presentation

A 79-year-old woman presented to the emergency department at a regional hospital with feculent emesis and inability to pass stool. Her medical history includes arterial hypertension and a hysterectomy 25 years prior. On physical examination, the patient was hemodynamically stable but dehydrated, with a soft, non-distended, epigastrically tender abdomen and reduced bowel sounds. A nasogastric tube was inserted, aspirating about 500 ml of gastric content. The CT findings were consistent with Rigler’s triad of pneumobilia, distended stomach, and ectopic gallstones, supporting the diagnosis of Bouveret’s syndrome. Emergent endoscopy was attempted. A Dormia basket was used to attempt stone extraction, but the large stone became impacted in the distal esophagus, causing a transmural perforation. The procedure was aborted, and the patient was transferred to our tertiary care center for further treatment.

**Figure 1:**
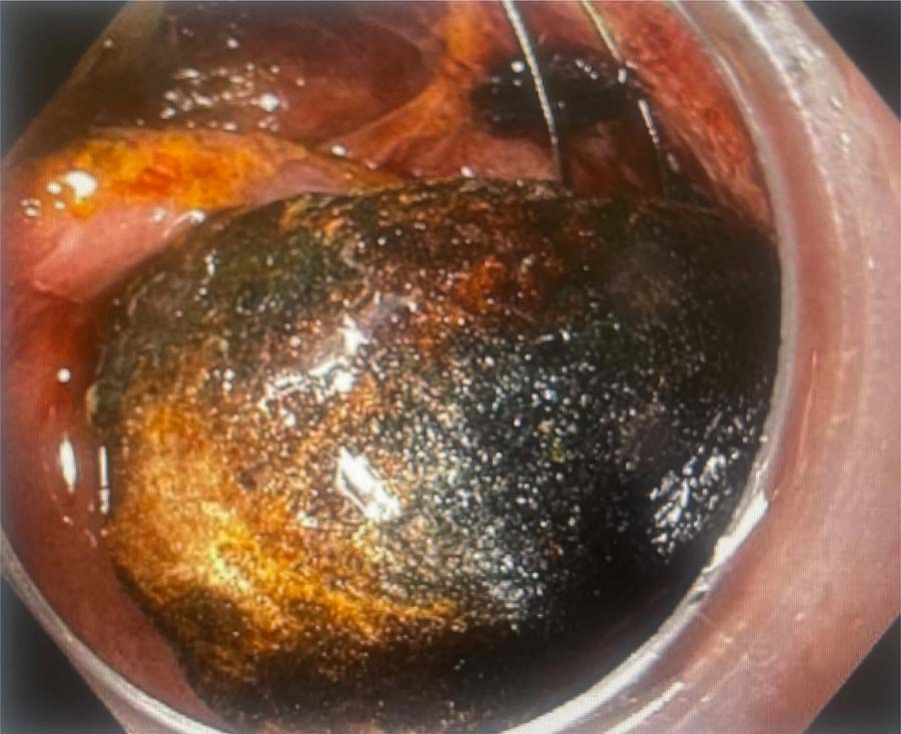
Gallstone trapped in a severed Dormia basket, with unsuccessful endoscopic retrieval.

**Figure 2:**
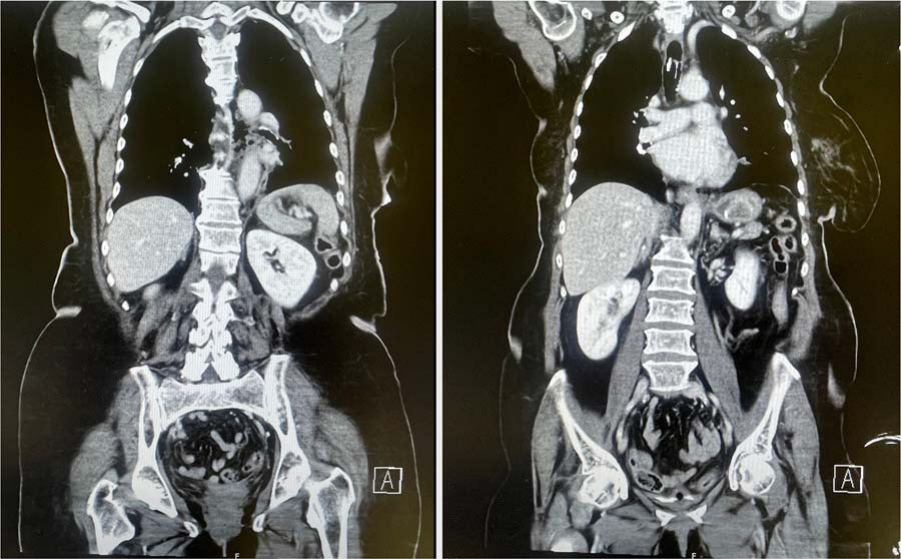
CT scan showing mediastinal air consistent with esophageal perforation (left) and a hyperdense structure in the stomach corresponding to the gallstone within the Dormia basket (right).

After repeat endoscopy (Fig. 1) and CT (Fig. 2) confirmed a 10 cm perforation with mediastinal air, emergency surgery was performed. A cervical-abdominal approach was used to resect the perforated esophagus and relieve the obstruction. Intraoperatively, extensive adhesions and a cholecystoduodenal fistula were found. Jejunal concretions and the impacted stone at the ligament of Treitz was removed. En bloc resection of the gallbladder and cholecystoduodenal fistula was performed and the duodenal defect was closed in two layers. A T-drain was inserted into the common bile duct and a percutaneous endoscopic gastrostomy tube was introduced for enteral feeding. The esophagus was circumferentially mobilized via a transhiatal approach and resected in discontinuity up to the esophagogastric junction, with the proximal esophagus exteriorized as a cervical esophagostomy. Postoperatively, the patient was transferred from the intensive care unit to the general ward on postoperative day three. All intraoperatively placed drains were removed in a timely manner due to unremarkable output. The postoperative course was otherwise unremarkable, and the patient was released into rehabilitative care.

Three months later, after clinical stabilization and nutritional optimization, the patient was readmitted for restoration of gastrointestinal continuity. A retrosternal colon interposition was chosen for reconstruction, as the stomach appeared too short for a gastric pull-up. The colon interposition, pedicled with adequate vascular supply, was transposed through the thorax for reconstruction with cervical esophagocolostomy an distal cologastrostomy. Continuity of the colon was reestablished and a percutaneous endoscopic jejunostomy was placed for postoperative enteral nutrition. The patient recovered well, gradually transitioned from enteral to oral feeding over 2 weeks, and was discharged home in good condition tolerating a soft diet, without complications.

## Discussion

### Endoscopic management of Bouvert’s syndrome

In accordance with current recommendations, our patient initially underwent endoscopic intervention for gallstone retrieval. Several cases have been reported in which even large gallstones were successfully retrieved endoscopically through the use of various lithotripsy techniques [6]. Nonetheless, the success rate remains low for stones larger than 2.5 cm, and life threatening complications such as perforation, bleeding, stone migration or even cardiac arrest may occur [1, 7]. Complete stone removal is crucial to prevent distal small bowel obstruction caused by residual fragments [8]. In our case, the gallstone measured more than 3 cm in diameter and endoscopic manipulation resulted in esophageal perforation. When endoscopic intervention proves difficult or unfeasible, early conversion to a surgical strategy should be considered. Endoscopic therapy demonstrates a substantially lower success rate of 37%–43% compared with surgical management (94%), with ~60% of surgeries performed as second-line treatment following failed endoscopy [9].

### Management of esophageal perforation

In our case, endoscopic manipulation resulted in a devastating complication: an iatrogenic distal esophageal perforation. Esophageal perforation is a life-threatening emergency associated with high mortality [10]. Therapeutic strategies include nonoperative management, endoscopic therapies and surgical repair, depending on the size, location and extent of contamination. According to the World Society of Emergency Surgery guidelines, nonoperative management is only suitable in selected patients with early diagnosis, clinical stability, minimal contamination, no underlying esophageal pathology and lesions suitable for endoscopic treatment [11]. In our case, the perforation was large, semicircumferential, and associated with mediastinal contamination, necessitating urgent surgical intervention. Surgical management included enterolithotomy for gallstone removal, cholecystectomy, fistula closure and esophageal resection, with delayed reconstruction of the continuity. For esophageal reconstruction, the most common methods include gastric pull-up or colon interposition [12]. Due to the excessive length of the required conduit in our case, gastric pull-up was not possible and a colon interposition was selected. While this technique allows full thoracic or even cervical esophageal replacement, it is associated with complications like anastomotic strictures (6%–2%), fistula formation (9%–40%), and medical morbidity (10%–75%) [12]. Despite the risks, his was the best viable option for our patient. She experienced an uneventful recovery, with restored swallowing function and nutritional status.

## Conclusion

Bouveret’s syndrome represents an exceedingly rare but critical condition and if complicated by iatrogenic esophageal perforation requires rapid diagnosis, interdisciplinary management, and tailored surgical intervention. Endoscopic retrieval remains the initial therapy of choice in many cases of Bouveret’s syndrome; however, caution must be exercised, particularly with large, impacted stones, due to the risk of catastrophic complications such as perforation. Cervical-abdominal esophageal discontinuity resection followed by delayed reconstruction, such as retrosternal colon interposition, offers a reliable and effective strategy for restoring continuity in such complex cases. This case highlights the need for extreme caution during endoscopic manipulation of large, impacted gallstones. Bouveret’s syndrome requires careful planning and close collaboration between gastroenterologists, radiologists, surgeons, anesthesiologists, and critical care specialists to optimize outcomes in elderly, multimorbid patients.
